# The Transcriptional Repressive Activity of KRAB Zinc Finger Proteins Does Not Correlate with Their Ability to Recruit TRIM28

**DOI:** 10.1371/journal.pone.0163555

**Published:** 2016-09-22

**Authors:** Kristin E. Murphy, Natalia A. Shylo, Katherine A. Alexander, Angela J. Churchill, Cecilia Copperman, María J. García-García

**Affiliations:** Department of Molecular Biology and Genetics, Cornell University, Ithaca, NY, United States of America; USDA-ARS, UNITED STATES

## Abstract

KRAB domain Zinc finger proteins are one of the most abundant families of transcriptional regulators in higher vertebrates. The prevailing view is that KRAB domain proteins function as potent transcriptional repressors by recruiting TRIM28 and promoting heterochromatin spreading. However, the extent to which all KRAB domain proteins are TRIM28-dependent transcriptional repressors is currently unclear. Our studies on mouse ZFP568 revealed that TRIM28 recruitment by KRAB domain proteins is not sufficient to warrant transcriptional repressive activity. By using luciferase reporter assays and yeast two-hybrid experiments, we tested the ability of ZFP568 and other mouse KRAB domain proteins to repress transcription and bind TRIM28. We found that some mouse KRAB domain proteins are poor transcriptional repressors despite their ability to recruit TRIM28, while others showed strong KRAB-dependent transcriptional repression, but no TRIM28 binding. Together, our results show that the transcriptional repressive activity of KRAB-ZNF proteins does not correlate with their ability to recruit TRIM28, and provide evidence that KRAB domains can regulate transcription in a TRIM28-independent fashion. Our findings challenge the current understanding of the molecular mechanisms used by KRAB domain proteins to control gene expression and highlight that a high percentage of KRAB domain proteins in the mouse genome differ from the consensus KRAB sequence at amino acid residues that are critical for TRIM28 binding and/or repressive activity.

## Introduction

Krüppel-associated box (KRAB) domain proteins are one of the most abundant protein families in higher vertebrates, with as many as 381 human and 357 mouse protein coding genes [1]. A remarkable feature of this protein family is that, with the exception of the MEISTZ protein in sea urchin, the KRAB motif is restricted to tetrapod organisms [2–5]. This limited distribution across the animal kingdom suggests that the KRAB motif emerged relatively recently in evolution, and that KRAB domain proteins expanded quickly in tetrapod organisms to perform functions specific to higher vertebrates [5]. Our understanding about the selective pressures leading to the expansion of this protein family is still hindered by our limited knowledge about the roles of individual members of this protein family. Nonetheless, research to date has made it clear that KRAB domain proteins have very diverse and specific biological functions. To date, KRAB domain proteins have been shown to regulate embryonic development, cell differentiation, ES cell maintenance, genomic imprinting, spermatogenesis, sex determination, cell signaling and retroviral silencing, to name a few (reviewed in [6]).

Because most KRAB domain-containing proteins contain a variable number of Zinc Finger motifs (ZNF) in their carboxyl terminal end [5], it has been proposed that KRAB-ZNF proteins function as transcriptional regulators, and that their diverse biological roles are dictated by their ability to recognize different DNA targets [7]. Support for this model has come from studies demonstrating that KRAB-ZNF proteins recognize specific DNA sequences through their ZNF motifs [8–10]. However, perhaps the most influential discovery towards understanding KRAB-domain protein function has been the finding that KRAB motifs can provide strong transcriptional repression [11,12]. KRAB-mediated repression has been shown to depend on the ability of the KRAB motif to interact with TRIM28 [13–15], a transcriptional co-repressor that functions by recruiting histone-modifying enzymes and promoting heterochromatin spreading [16–23]. Given that the majority of KRAB-ZNF proteins studied to date repress transcription [8,9,11,12,24] and that mutations in KRAB motif residues that disrupt TRIM28 binding also cause loss of transcriptional repression [13,25], the prevailing view has been that all KRAB domain proteins are TRIM28-dependent transcriptional repressors. However, this assumption has been challenged by a few studies reporting that some human KRAB domain proteins do not bind TRIM28 and have poor repressive activity [26], as well as by evidence that at least one human KRAB-ZNF protein can activate transcription [27].

In this study, we report the characterization of ZFP568 (NCBI Gene ID: 243905), a mouse KRAB-ZNF protein required for early embryonic morphogenesis [28,29]. Unlike most KRAB-ZNF proteins, ZFP568 contains two KRAB domains, a feature that prompted us to investigate how the first and second KRAB motifs of ZFP568 contribute to its overall activity. Our results show that the first and second KRAB motifs of ZFP568 have different contributions to ZFP568 transcriptional repressive activity and ability to bind TRIM28, and that their different properties are due to variations in the KRAB motif amino acid composition. Interestingly, our analysis of ZFP568 also suggests that TRIM28 recruitment by KRAB domain proteins is not sufficient to warrant transcriptional repressive activity. To further corroborate and expand these observations, we tested TRIM28 binding and transcriptional repression in a selection of mouse KRAB domain proteins. Our findings highlight that a significant fraction of KRAB domain proteins in the mouse genome are poor transcriptional repressors, despite their ability to recruit TRIM28. Conversely, we found that some KRAB domain proteins are unable to recruit TRIM28, yet they are potent transcriptional repressors. Together, our results challenge the current understanding of the roles of KRAB motifs by showing that the transcriptional repressive activity of KRAB-ZNF proteins does not correlate with their ability to recruit TRIM28, and by providing evidence that the KRAB motif can repress transcription through TRIM28-independent mechanisms.

## Results

### The *chato* mutation disrupts the repressive activity of ZFP568

**Fig 1:**
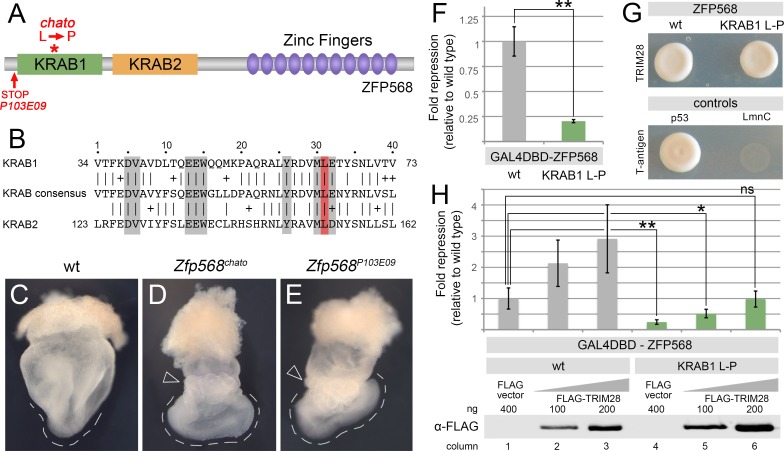
The *chato* mutation disrupts ZFP568 repressive activity, but not its ability to bind TRIM28. **(A)** Domain structure of ZFP568. The red asterisk indicates the location of the *chato* mutation, which causes a Leu to Pro substitution. The red arrow indicates the position of the early stop codon in *P103E09* mutants, which results in a truncated protein containing only the first ZFP568 11 amino acids. **(B)** Sequence alignment of the first and second KRAB motifs of ZFP568 with the Pfam KRAB consensus. Grey boxes indicate residues important for repressive activity as described in [11–13,25]. The red box highlights the conserved Leu residue mutated in *chato*. **(C-E)** Whole mount wild type (C), *Zfp568*^*chato*^ (D) and *Zfp568*^*P103E09*^ (E) E8.5 embryos. The discontinued line highlights the abnormal U-shape of *Zfp568* mutants, as compared with the V-shaped profile of wild type embryos. Empty arrowheads point to the yolk sac, which is abnormally ruffled in *Zfp568* mutants. **(F)** Quantification of luciferase expression from a 5xUAS-luciferase reporter in HEK293T cells, in the presence of wild type (grey) and a KRAB1 L-P mutant (green) GAL4DBD-ZFP568. **(G)** Yeast two-hybrid assay for TRIM28 interaction with wild type ZFP568 (upper left) and a KRAB1 Leu-Pro mutant ZFP568 (upper right). T-antigen and p53 interaction was used as positive control. T-antigen and LmnC were used as negative control. **(H)** Quantification of luciferase expression from a 5xUAS-luciferase reporter in HEK293T cells, in the presence of wild type (grey) and KRAB1 Leu-Pro mutant (green) GAL4DBD-ZFP568, either in the absence (columns 1, 4) or presence of increasing amounts of FLAG-TRIM28 (columns 2–3 & 5–6). Luciferase expression is graphed as fold repression normalized to Gal4DBD (empty vector) and relative to wild type (column 1). Error bars represent standard deviation. Western blots show levels of FLAG-TRIM28 protein for each condition. Asterisks indicate samples which pairwise comparison had a p<0.05 (*) or p<0.005 (**). ns, no statistical significance.

A previous forward mutagenesis screen [30] identified *chato*, a recessive lethal mutation in the mouse KRAB domain protein ZFP568 [28]. The *chato* mutation causes a Leucine to Proline (L64P) amino acid substitution in a conserved residue of the first KRAB motif of ZFP568 (Fig 1A and 1B). Homozygote mutant embryos for either the *Zfp568*^*chato*^ alelle or a null allele of *Zfp568* (*Zfp568*^*P103E09*^) show similar developmental defects, including abnormal convergent extension and disrupted morphogenesis of extraembryonic tissues (Fig 1C–1E; [28,29]). Additionally, the phenotype of transheterozygote *Zfp568*^*chato/P103E09*^ embryos is indistinguishable from either *Zfp568*^*chato*^ or *Zfp568*^*P103E09*^ mutants, indicating that the *chato* L64P point mutation severely disrupts ZFP568 function [28].

Previous mutagenesis studies of KRAB domain proteins showed that the Leucine residue affected by the *chato* mutation is important for KRAB-mediated repression. Specifically, MLE-to-AAA or MLE-to-KKK mutations in the amino acids 30 to 32 of the KRAB consensus sequence cause loss of repressive activity (S1 Fig; [11]) and disrupt the ability of the KRAB motif to recruit the transcriptional co-repressor TRIM28 (S1 Fig; [13,25]). Consequently, we hypothesized that the *chato* L64P point mutation disrupts ZFP568 function due to loss of repressive activity and/or failure to bind TRIM28. To test this hypothesis, we used a mammalian luciferase reporter system and yeast two-hybrid assays. Transfection of a GAL4DBD-ZFP568 chimeric construct in HEK293T cells reduced the transcription level of a UAS-luciferase reporter by an average of 84% as compared with cells transfected with a GAL4DBD construct, confirming that ZFP568 is a potent transcriptional repressor. In contrast, a mutant GAL4DBD-ZFP568 version containing the *chato* L64P mutation (GAL4DBD-ZFP568^KRAB1L-P^) severely impaired the repressive activity of GAL4DBD-ZFP568 (Fig 1F). These results indicate that the conserved Leucine mutated in the *chato* allele is critical for ZFP568 function and KRAB-mediated transcriptional repression.

We anticipated that the reduced transcriptional repressive activity of GAL4DBD-ZFP568^KRAB1L-P^ would likely be due to loss of TRIM28 binding. However, a GAL4DBD-ZFP568^KRAB1L-P^ mutant construct was able to interact with TRIM28 in yeast two-hybrid assays (Fig 1G). Consistent with the ability of GAL4DBD-ZFP568^KRAB1L-P^ to recruit TRIM28, we found that the repressive activity of GAL4DBD-ZFP568^KRAB1L-P^ in luciferase assays was enhanced when ectopic TRIM28 was transfected into cells (Fig 1H). Therefore, even though the *chato* mutation severely disrupts the transcriptional repressive activity of ZFP568, our results show that the L64P mutation does not abolish its ability to recruit TRIM28.

### The first and second KRAB domains of ZFP568 have different repressive activities

**Fig 2:**
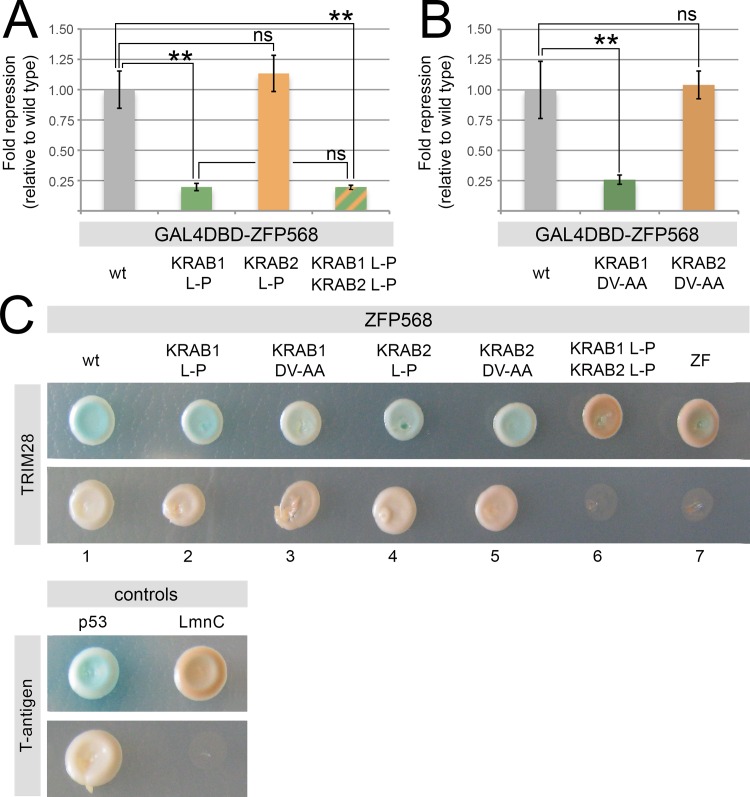
Mutations in the second KRAB motif of ZFP568 do not disrupt ZFP568 repressive activity. **(A-B)** Quantification of luciferase expression from a 5xUAS-luciferase reporter in HEK293T cells, in the presence of wild type (grey) and mutant versions of GAL4DBD-ZFP568 as follows: KRAB1 L-P (green in A), KRAB2 L-P (orange in A), L-P mutations in both KRAB1 & KRAB2 (striped), KRAB1 DV-AA (green in B), KRAB2 DV-AA (orange in B). **(C)** Yeast two-hybrid assay testing the interaction of TRIM28 with wild type and mutant versions of ZFP568 as indicated. T-antigen and p53 interaction was used as positive control. T-antigen and LmnC were used as negative control. A truncated ZFP568 lacking all KRAB motifs (ZF) was also used as a negative control (column 7). The pictures show representative yeast colonies for each of the interactions tested, as grown on low stringency media (upper row) and high stringency media (lower row). Luciferase expression is graphed as fold repression normalized to Gal4DBD (empty vector) and relative to wild type. Error bars represent standard deviation. Asterisks indicate samples which pairwise comparison had a p<0.05 (*) or p<0.005 (**). ns, no statistical significance.

Because ZFP568 has two KRAB motifs, we investigated whether the second ZFP568 KRAB domain can compensate for the effects of the *chato* L64P mutation and provide residual repressive activity and TRIM28 binding in *Zfp568*^*chato*^ mutants. To resolve the respective contributions of the first and second KRAB motifs of ZFP568, we performed mammalian luciferase reporter and yeast two-hybrid assays using versions of ZFP568 with mutations in its first, second or both KRAB domains. We found that while the L64P mutation in the first KRAB domain of ZFP568 significantly decreased the repressive activity of GAL4DBD-ZFP568 in luciferase assays (green bar, Fig 2A), the equivalent L-P mutation in the second ZFP568 KRAB motif (L153P) had no significant effect on transcriptional repression as compared with wild type GAL4DBD-ZFP568 (orange bar, Fig 2A). Moreover, the repressive activity of a GAL4DBD-ZFP568 mutant with L-P mutations in both the first and second KRAB motifs (KRAB1 L-P, KRAB2 L-P, striped bar in Fig 2A) was similar to that of the GAL4DBD-ZFP568 mutant in the first KRAB motif (green bar, Fig 2A). These results suggest that the first and second KRAB motifs of ZFP568 have different contributions to the repressive activity of ZFP568. To further confirm this observation, we tested whether mutations in different amino acid residues of the ZFP568 KRAB motifs had similar effects on transcriptional activity. We introduced DV to AA mutations in residues 5–6 of the consensus KRAB sequence, since these mutations were previously reported to disrupt both KRAB-mediated transcriptional repression and TRIM28 binding [1,11–13,25]. Similar to L-P mutations, we found that DV-AA mutations in the first KRAB motif of ZFP568 caused a significant decrease in the transcriptional repressive activity of GAL4DBD-ZFP568, but DV-AA mutations in the second ZFP568 KRAB motif did not affect transcriptional repression (Fig 2B, compare with Fig 2A). Therefore, our analysis of ZFP568 mutants in luciferase assays indicates that the second KRAB motif of ZFP568 is dispensable for ZFP568 transcriptional repressive activity and that transcriptional repression by ZFP568 is entirely carried out by the first KRAB motif.

In contrast to the different contributions of ZFP568 first and second KRAB domains to its transcriptional repressive activity, yeast two-hybrid assays indicated that both KRAB motifs contribute to ZFP568 ability to recruit TRIM28. Specifically, we found that L-P and DV-AA mutations in either the first or the second KRAB motif did not eliminate the ability of ZFP568 to bind TRIM28 (Fig 2C, columns 1–5), but that a GAL4DBD-ZFP568 construct carrying mutations in both KRAB motifs failed to bind TRIM28 in yeast two-hybrid experiments (Fig 2C, column 6). Together, these results are consistent with previous evidence supporting that TRIM28 binding is a pre-requisite for the repressive activity of KRAB domain proteins [13,25]. Furthermore, our finding that the second KRAB motif of ZFP568 is dispensable for transcriptional repression, despite contributing to TRIM28 recruitment, revealed that the ability of KRAB domain proteins to recruit TRIM28 is not sufficient for their transcriptional repressive activity.

### A threshold of TRIM28 binding is required for KRAB-mediated transcriptional repression

**Fig 3:**
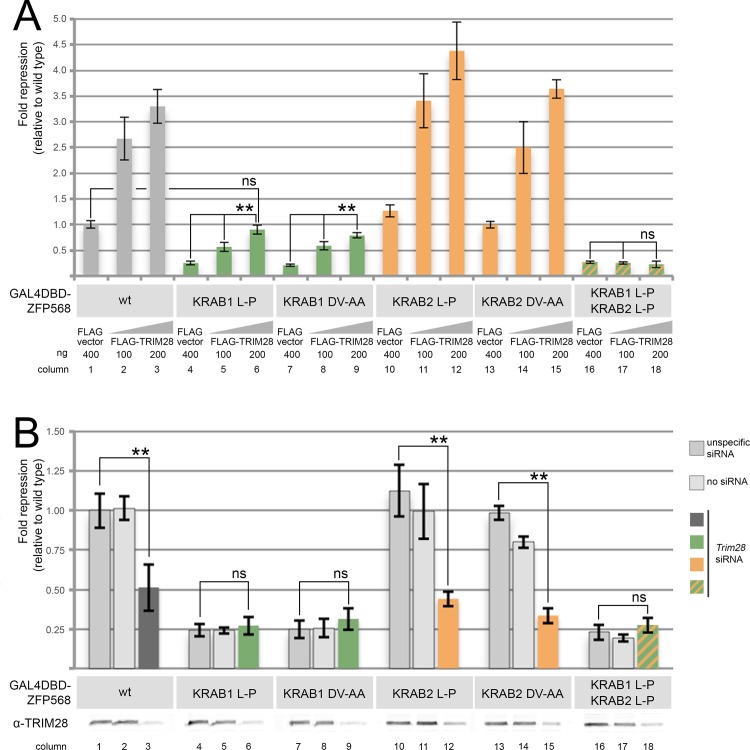
Mutations in both the first and second KRAB motifs of ZFP568 affect ZFP568 ability to respond to TRIM28 levels. Quantification of luciferase expression from a 5xUAS-luciferase reporter in HEK293T cells, in the presence of wild type and mutant versions of GAL4DBD-ZFP568 as indicated. **(A)** Luciferase expression in the presence of increasing amounts of transfected FLAG-TRIM28. **(B)** Luciferase expression upon TRIM28 depletion with siRNA. No siRNA and unspecific siRNAs were used as controls. Western blots show levels of TRIM28 as tested with α-TRIM28 antibodies. Luciferase expression is graphed as fold repression normalized to Gal4DBD (empty vector) and relative to wild type (column 1). Error bars represent standard deviation. Asterisks indicate samples which pairwise comparison had a p<0.005 (**). ns, no statistical significance.

We speculated whether the different repressive activities of the first and second KRAB motifs of ZFP568 could be due to differences in their ability to recruit TRIM28. To investigate this possibility, we used our mammalian luciferase reporter assay to test the responsiveness of ZFP568 mutants to changes in the amount of TRIM28. We found that GAL4DBD-ZFP568^KRAB1L-P, KRAB2L-P^ was unresponsive to transfection of increasing amounts of TRIM28 (Fig 3A, columns 16–18), a result consistent with our previous finding that this ZFP568 mutant is unable to bind TRIM28 (Fig 2C, column 6). In contrast, we found that the transcriptional repressive activities of ZFP568 mutants that retained TRIM28 binding (GAL4DBD-ZFP568^KRAB1L-P^, GAL4DBD-ZFP568^KRAB2L-P^, GAL4DBD-ZFP568^KRAB1DV-AA^ and GAL4DBD-ZFP568^KRAB2DV-AA^) all increased in response to ectopically expressed TRIM28 (Fig 3A, columns 1–15). Interestingly, when TRIM28 was transfected, transcriptional repression by GAL4DBD-ZFP568^KRAB1L-P^ reached levels that were not significantly different from those of wild type ZFP568 in cells without ectopic TRIM28 (Fig 3A, compare columns 1 and 6). The fact that increasing the total concentration of TRIM28 in cells can influence the levels of KRAB-mediated transcriptional repression suggests that the relative repressive activity of KRAB domain proteins greatly depends on the affinity of their KRAB motifs to recruit TRIM28.

To further investigate the responsiveness of ZFP568 mutants to TRIM28 levels, we tested their repressive activity in luciferase reporter assays when TRIM28 was knocked down using siRNAs. In these experiments, we found that transcriptional repression by wild type ZFP568, as well as ZFP568 mutants in the second KRAB motif (GAL4DBD-ZFP568^KRAB2L-P^ and GAL4DBD-ZFP568^KRAB2DV-AA^) significantly decreased when TRIM28 siRNAs were transfected (Fig 3B, columns 1–3 and 10–15). In contrast, ZFP568 mutants in the first KRAB motif (GAL4DBD-ZFP568^KRAB1L-P^ and GAL4DBD-ZFP568^KRAB1DV-AA^) or in both the first and second KRAB domains (GAL4DBD-ZFP568^KRAB1L-P, KRAB2L-P^) failed to respond to the decrease in TRIM28 caused by siRNAs (Fig 3B, columns 4–9 and 16–18). These results corroborate our previous observations that the two KRAB motifs in ZFP568 have different transcriptional repressive activity. Additionally, since ZFP568 mutants in the first KRAB motif increased their repressive activity in response to ectopic TRIM28, but did not respond to loss of TRIM28 function, our experiments suggest that a certain threshold of TRIM28 binding is required for KRAB motifs to efficiently repress transcription in vivo.

### Transcriptional repression by KRAB domain proteins does not depend on the relative position or number of KRAB motifs

**Fig 4:**
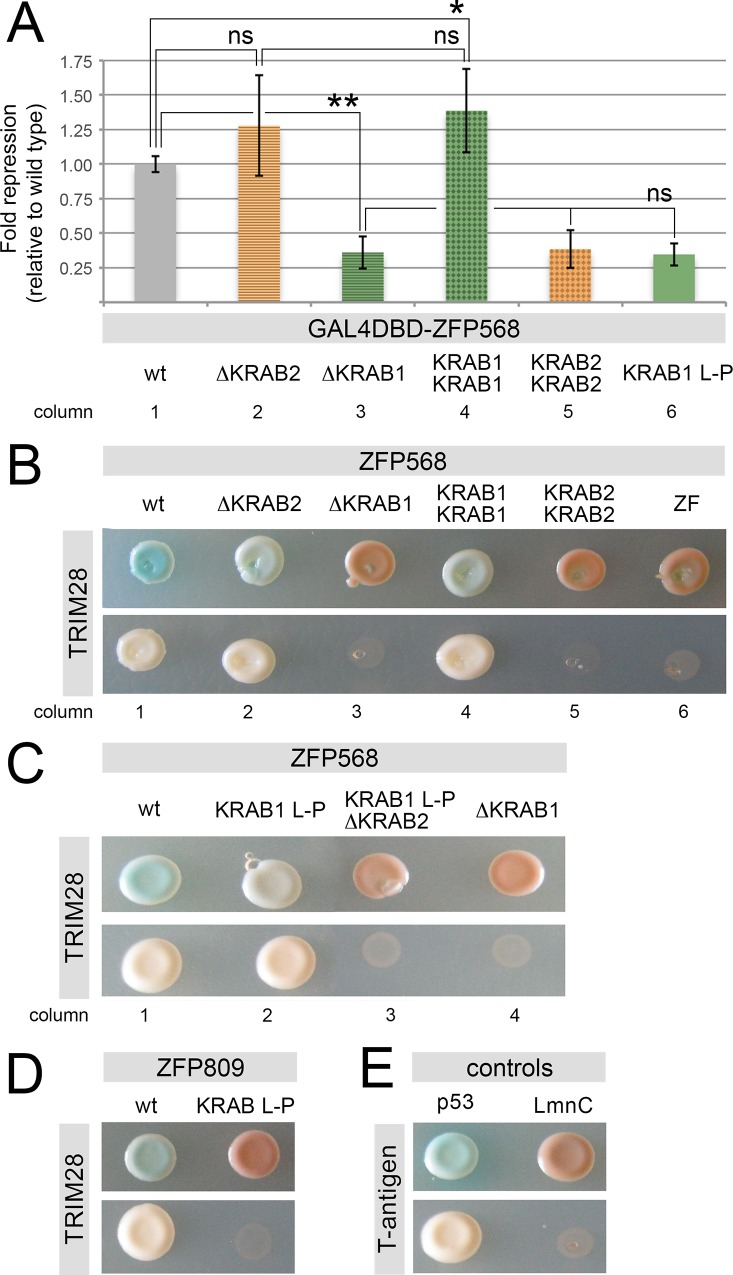
The repressive activity of ZFP568 depends on the properties of its KRAB motifs, but not on their number or location within the protein. **(A)** Quantification of luciferase expression from a 5xUAS-luciferase reporter in HEK293T cells, in the presence of wild type (grey) and mutant versions of GAL4DBD-ZFP568: lacking the second KRAB motif (∆KRAB2), lacking the first KRAB motif (∆KRAB1), with two KRAB1 motifs (KRAB1 KRAB1), with two KRAB2 motifs (KRAB2 KRAB2), or with a mutated KRAB1 motif (KRAB1 L-P). Luciferase expression is graphed as fold repression compared to Gal4DBD (empty vector) and relative to wild type (column 1). Error bars represent standard deviation. **(B-D)** Yeast two-hybrid assays showing the interaction of TRIM28 with wild type and mutant versions of ZFP568 and ZFP809 as indicated. **(E)** T-antigen and p53 were used as positive controls. T-antigen and LmnC were used as negative controls. A truncated ZFP568 lacking all KRAB motifs (ZF) was also used as a negative control in panel B (column 6). The pictures show representative yeast colonies for each of the interactions tested, as grown on low stringency media (upper row) and high stringency media (lower row). Asterisks indicate samples which pairwise comparison had a p<0.05 (*) or p<0.005 (**). ns, no statistical significance.

We sought to determine whether the differences in repressive activity between ZFP568 first and second KRAB motifs are due to their relative positions within the protein or to their inherent properties. To this end, we first tested the transcriptional repressive activity and TRIM28 binding in mutant versions of ZFP568 that lacked the entire first or second KRAB motifs. We found that deletion of the first KRAB motif significantly reduced the repressive activity of ZFP568 in luciferase reporter assays (Fig 4A, columns 1 & 3), but that deletion of the second KRAB motif did not have a significant effect on the transcriptional repressive activity of ZFP568 (Fig 4A, columns 1–2). The relative effects of deletions in the first and second KRAB motifs of ZFP568 were similar to those we previously observed when we introduced L-P and DV-AA mutations, since only disruptions in the first KRAB motif caused significant alterations in the transcriptional repressive activity of ZFP568 (Fig 2A and 2B & Fig 4A, columns 1–3 and 6). Consequently, our results show that the different transcriptional repressive activity of the first and second KRAB motifs of ZFP568 does not depend on their position within the protein.

Interestingly, we found that the effects of KRAB domain deletions on the ability of ZFP568 to recruit TRIM28 were different than those we had previously observed for point mutations in ZFP568 KRAB motifs. Specifically, we found that TRIM28 failed to bind ZFP568 in yeast two-hybrid experiments when the first KRAB motif was completely deleted (ZFP568^∆KRAB1^; Fig 4B, column 3), suggesting that the second KRAB domain of ZFP568 is unable to recruit TRIM28 on its own. This result contrasts with our previous finding that TRIM28 binds to ZFP568^KRAB1L-P^ mutants (Fig 2C, columns 2–3), a result that we attributed to the ability of ZFP568 second KRAB motif to recruit TRIM28. We hypothesized that this discrepancy in TRIM28 binding between ZFP568^∆KRAB1^ and ZFP568^KRAB1L-P^ mutants could be due to an ability for the second KRAB motif of ZFP568 to complement the effects of the ZFP568^KRAB1L-P^ mutation. In order to test this hypothesis, we first re-evaluated the effects of KRAB domain L-P mutations on TRIM28 binding by performing yeast two-hybrid assays using wild type and L-P mutant versions of ZFP809, a KRAB-ZNF protein involved in retroviral silencing that contains a single KRAB motif [9]. We found that the L-P mutation disrupted the ability of ZFP809 to recruit TRIM28 (Fig 4D), providing further evidence that this Leu residue is important for TRIM28 binding, and suggesting that TRIM28 does not bind the first KRAB motif of ZFP568 in ZFP568^KRAB1L-P^ mutants. We next addressed whether the second KRAB motif of ZFP568 contributes to recruit TRIM28 in ZFP568^KRAB1L-P^ mutants by testing whether a ZFP568^KRAB1L-P^ mutant construct could still recruit TRIM28 when the second KRAB motif was deleted (ZFP568^KRAB1L-P; ∆KRAB2^ mutant). As shown in Fig 4C, ZFP568^KRAB1L-P; ∆KRAB2^ mutants failed to recruit TRIM28 in yeast two-hybrid experiments. Therefore, although the second KRAB motif of ZFP568 could not bind TRIM28 on its own (Fig 4B column 3, Fig 4C column 4), these results suggest that the second KRAB motif of ZFP568 can compensate for the inability of the KRAB1 L-P mutated motif to bind TRIM28. Together with our previous finding that TRIM28 fails to bind ZFP568 when L-P mutations are introduced in the first and second motifs of ZFP568 (Fig 2C, column 6), our data suggest that the first and second KRAB motifs of ZFP568 cooperate to recruit TRIM28.

To test if the presence of more than one KRAB motif can elicit a cooperative effect in recruiting TRIM28 and enhancing the repressive activity of KRAB-containing proteins, we tested the repressive activity and TRIM28 binding ability of engineered versions of ZFP568 that contained either two KRAB1 motifs or two KRAB2 motifs. The presence of two KRAB1 motifs enhanced the transcriptional repressive activity of ZFP568 as compared with wild type ZFP568 (Fig 4A, compare columns 1 & 4; p = 0.045), but the transcriptional repression of ZFP568^KRAB1-KRAB1^ was not statistically different compared to that of the ZFP568^∆KRAB2^ construct (Fig 4A, compare columns 2 & 4). Most interestingly, the presence of two KRAB2 motifs did not enhance the repressive activity of ZFP568 as compared with those of ZFP568^KRAB1L-P^ and ZFP568^∆KRAB1^ constructs (Fig 4A, columns 3, 5–6), nor was the engineered ZFP568 with two KRAB2 motifs able to recruit TRIM28 in yeast two-hybrid assays (Fig 4B, column 5). Therefore, we conclude that, although the presence of more than one KRAB motif can slightly enhance the repressive activity of KRAB-ZNF proteins, it is the inherent repressive ability of their KRAB motifs, and not their number, which determines the overall repressive activity of KRAB domain proteins.

### Transcriptional repression by KRAB domain proteins depends on critical amino acid residues

**Fig 5:**
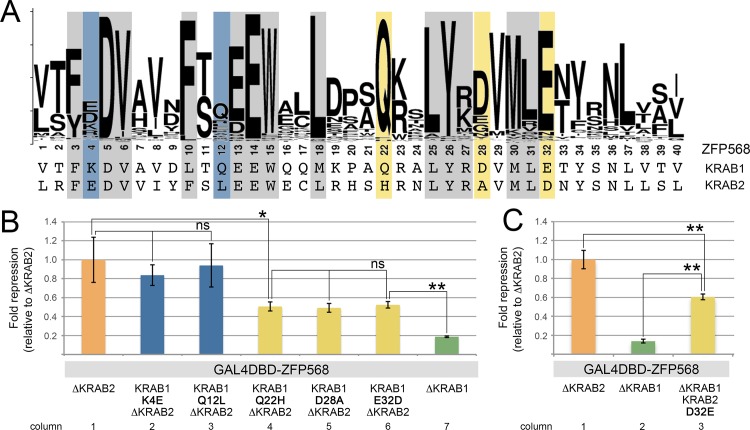
Amino acid differences between the first and second KRAB motifs of ZFP568 influence transcriptional repressive activity. **(A)** Alignment of the amino acid sequence of the first and second KRAB domains of ZFP568 with a weblogo for all mouse KRAB domains. Residues highlighted in grey indicate residues important for repressive activity as described in [11–13,25]. Residues highlighted in blue and yellow indicate differences between the first and second ZFP568 KRAB motifs that were tested for their effects on transcriptional repressive activity as shown in B and C. (**B-C)** Quantification of luciferase expression from a 5xUAS-luciferase reporter in HEK293T cells in the presence of **(B)** mutant versions of GAL4DBD-ZFP568 lacking the second KRAB motif and with KRAB1-to-KRAB2 amino acid substitutions in the indicated KRAB1 domain residues (K4E in column 2, Q12L in column 3, Q22H in column 4, D28A in column 5 & E32D in column 6) and **(C)** mutant versions of GAL4DBD-ZFP568 lacking the first KRAB motif and with KRAB2-to-KRAB1 amino acid substitutions in the indicated KRAB2 domain residues (D32E in column 3). Mutant versions of GAL4DBD-ZFP568 lacking the second KRAB motif (∆KRAB2, column 1) or the first KRAB motif (∆KRAB1, column 7 in B, column 2 in C) are shown for comparison. Luciferase expression is graphed as fold repression compared to Gal4DBD (empty vector) and relative to ∆KRAB2 (column 1). Error bars represent standard deviation. Asterisks indicate samples which pairwise comparison had a p<0.05 (*) or p<0.005 (**). ns, no statistical significance.

To further investigate whether differences in the amino acid sequence of the KRAB motif are critical for its transcriptional repression activity, we tested whether substituting individual amino acids in the first ZFP568 KRAB motif for those at the equivalent position in the second ZFP568 KRAB domain affects repressive activity. In order to isolate possible cooperative effects of the two ZFP568 KRAB motifs, we used the GAL4DBD-ZFP568^∆KRAB2^ construct as the baseline control for these experiments. Site-directed mutagenesis was performed on selected residues that were different between the two ZFP568 KRAB motifs and/or represented non-conservative amino acid substitutions (Fig 5A). We found that K4E and Q12L substitutions in the first ZFP568 KRAB motif did not cause significant alterations in repressive activity (Fig 5A and 5B, blue). However, Q22H, D28A and E32D substitutions produced a significant reduction in transcriptional repression (Fig 5A and 5B, yellow), identifying specific KRAB domain residues previously unknown to contribute to KRAB-mediated repressive activity (see S1 Fig).

While the repressive activity of the first ZFP568 motif was substantially reduced by Q22H, D28A and E32D substitutions, repression levels were higher than those driven by the second ZFP568 KRAB motif (Fig 5B, compare columns 4, 5 and 6 with column 7). Additionally, we found that a reciprocal D32E substitution in the second ZFP568 KRAB motif caused an increase in repressive activity (Fig 5C, compare columns 2 and 3), but did not reach the high level of repression characteristic of the first ZFP568 KRAB motif (Fig 5C, compare columns 3 and 1). Consequently, we conclude that the difference in repressive activity between the first and second KRAB motifs of ZFP568 is likely due to amino acid differences at multiple residues.

### Transcriptional repression by KRAB domain proteins does not correlate with their ability to recruit TRIM28

**Fig 6:**
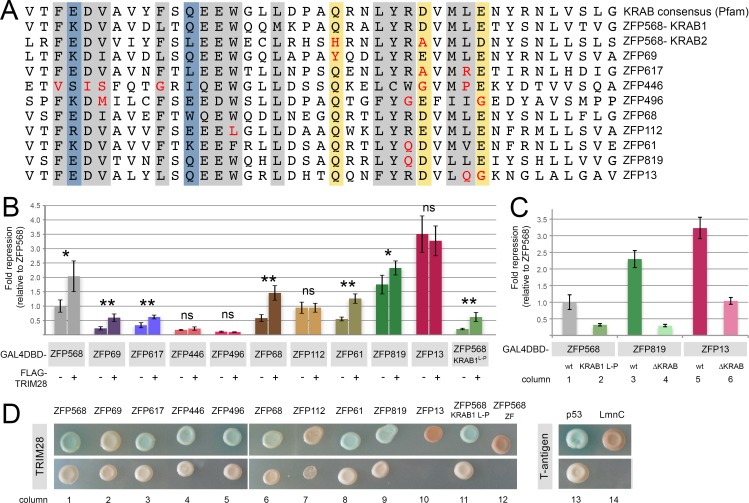
Transcriptional repression and TRIM28 binding in selected mouse KRAB Zinc finger proteins. **(A)** Alignment of KRAB domain sequences of ZFP568 with the indicated mouse KRAB domain proteins. Residues highlighted in grey indicate amino acids important for repressive activity (as described in [11–13,25]). Amino acid differences between the first and second ZFP568 KRAB motifs are highlighted in yellow, if they showed an impact in transcriptional repressive activity, or in blue, if they had no impact. Red font was used to highlight critical KRAB residues that constitute non-conservative amino acid substitutions with respect to the KRAB consensus sequence and/or are not highly represented in mouse KRAB domain proteins. **(B-C)** Quantification of luciferase expression from a 5xUAS-luciferase reporter in HEK293T cells for the indicated mouse Zinc finger proteins, **(B)** in the absence (-) or presence (+) of transfected FLAG-TRIM28. **(C)** in wild type and mutant versions that carried point mutations or lacked the KRAB motif as indicated. Luciferase expression is graphed as fold repression compared to Gal4DBD (empty vector) and relative to wild type ZFP568. Error bars represent standard deviation. Asterisks indicate samples which pairwise comparison had a p<0.05 (*) or p<0.005 (**). ns, no statistical significance. **(D)** Yeast two-hybrid assays showing the interaction of TRIM28 with the indicated mouse KRAB domain proteins. ZFP568 L-P mutant was included for reference. T-antigen and p53 were used as positive controls. T-antigen and LmnC were used as negative controls. A truncated ZFP568 lacking all KRAB motifs was also used as a negative control (column 12). The pictures show representative yeast colonies for each of the interactions tested, as grown on low stringency media (upper row) and high stringency media (lower row).

By examining an alignment of all KRAB motifs in the mouse genome, we noticed that a large number of KRAB domain-containing proteins differ from the consensus KRAB motif sequence at amino acid residues that we and others have found to be important for transcriptional repression and TRIM28 binding (S1 Table worksheets #3 & #4; [11–13,25]). This observation suggests that a significant percentage of KRAB domain proteins lack repressive activity. To investigate whether this is the case, we selected a subset of mouse KRAB domain proteins and checked their ability to repress transcription in luciferase reporter assays. We selected proteins in which critical sequences (grey, blue and yellow shaded areas in Fig 6A) contain residues that are not highly represented in KRAB domain proteins (see Fig 5A logo) and/or that constitute non-conservative amino acid substitutions as compared with the consensus KRAB domain sequence (Fig 6A, see residues highlighted in red). We found that some of these selected KRAB domain proteins, including ZFP69, ZFP617, ZFP446 and ZFP496, had poor repressive activity in luciferase assays (Fig 6B), a result that is consistent with their divergent amino acid sequence at critical residues. However, some other selected proteins (ZFP112, ZFP819 and ZFP13) could repress transcription to similar or higher levels than ZFP568 (Fig 6B). Especially surprising was the high repressive activity of ZFP13, since this protein contained non-conservative amino acid substitutions at the conserved MLE consensus sequence (Fig 6A). We wondered whether the high repressive activity of these proteins could be due to residues outside of their KRAB motifs. Search engines failed to identify any known repressive domains in ZFP819 and ZFP13 besides the KRAB region (S2 Fig). Nonetheless, to directly test the presence of repressive motifs in these proteins, we analyzed the repressive activity of ZFP819 and ZFP13 mutants lacking their KRAB domains. We found that deletion of the KRAB motif decreased the repressive activities of both ZFP819 and ZFP13 (Fig 6C), although ZFP13^∆KRAB^ showed a level of repression comparable to that of wild type ZFP568 (compare columns 6 and 1 in Fig 6C). While these experiments suggest that ZFP13 can repress transcription through KRAB-independent mechanisms, our results also show that the KRAB motifs in these proteins have substantial repressive activity, despite their divergent amino acid sequence at critical residues.

To evaluate whether the transcriptional repression by these selected KRAB domain proteins can be explained by their different ability to recruit TRIM28, we performed yeast two-hybrid experiments. We found that most proteins were able to recruit TRIM28, regardless of whether they had low or high repressive activity (Fig 6D). Most intriguingly, TRIM28 binding was reduced or absent in experiments with ZFP112 and ZFP13, two of the proteins with relatively high repressive activity (Fig 6D, columns 7 & 10). We sought to confirm whether our selected KRAB domain proteins could bind TRIM28 by testing their responsiveness to ectopic TRIM28 in luciferase reporter assays. With the exception of ZFP446 and ZFP496, which had very low repressive activity, these experiments confirmed that proteins showing TRIM28 binding in yeast two-hybrid experiments increased their transcriptional repressive activity in cells transfected with ectopic TRIM28 (Fig 6B). Reciprocally, transcriptional repression by ZFP112 and ZFP13 did not significantly change in response to ectopic TRIM28 (Fig 6B), a result that is consistent with their impaired ability to recruit TRIM28. Together with our previous experiments on ZFP568, our results reveal that transcriptional repression by KRAB domain proteins does not correlate with their ability to recruit TRIM28. First, our studies highlight that TRIM28 recruitment is not sufficient for transcriptional repression. Additionally, our results provide evidence that the KRAB domain can repress transcription through TRIM28-independent mechanisms.

## Discussion

Our studies provide insight about the molecular mechanisms of KRAB-mediated transcriptional repression by revealing that the repressive activity of the KRAB domain proteins does not correlate with their ability to recruit TRIM28. The first evidence supporting this conclusion comes from our studies on ZFP568, an atypical mouse KRAB domain protein containing two KRAB motifs. Using a luciferase reporter system and yeast two-hybrid assays, we found that mutations in the first KRAB motif of ZFP568 disrupt its repressive activity, but not its ability to recruit TRIM28, suggesting that TRIM28 recruitment is not sufficient for KRAB-domain mediated repression. Because this result challenges the current assumption that TRIM28 recruitment is necessary and sufficient for KRAB proteins to repress transcription [5,7,11–13,25], we investigated whether a certain level of TRIM28 recruitment is required to warrant transcriptional repression. Consistent with previous findings, we observed that the transcriptional repressive activity of ZFP568 can be impaired by reducing the amount of TRIM28 with siRNAs. More interestingly, we also found that the repressive activity of mutant versions of ZFP568 that are able to recruit TRIM28 can be potentiated by ectopic expression of TRIM28. Together, these results support the conclusion that TRIM28 recruitment is not sufficient for the transcriptional repressive activity of KRAB domain proteins, but that a certain TRIM28 threshold is required for the KRAB motif to repress transcription efficiently. Similar to previous studies on KRAB domain proteins, this conclusion relies on results from reporter assays, raising the question of whether results from reporter systems accurately represent the conditions that occur in vivo. In this regard, it is important to note that an L-P mutation in the first KRAB motif of ZFP568 causes similar embryonic defects in mice to those of null ZFP568 mutants, arguing that the decrease in transcriptional repression caused by the L-P mutation in our reporter assays is significant in vivo. Consequently, the affinity of KRAB domain proteins to bind TRIM28 is important to determine their biological activity.

Another important conclusion from our studies on ZFP568 was the realization that the first and second KRAB motifs of this atypical KRAB-ZNF protein have different contributions to its repressive activity and ability to recruit TRIM28. Specifically, we found that the first ZFP568 KRAB motif is important for transcriptional repression and TRIM28 recruitment, while the second ZFP568 KRAB motif has poor transcriptional repressive activity but still contributes to TRIM28 binding. An interesting aspect of our studies is the finding that the second KRAB motif of ZFP568 can not bind to TRIM28 by itself, but that it cooperates with the first ZFP568 KRAB motif, enhancing its ability to recruit TRIM28. Knowledge about KRAB-ZNF with two conserved KRAB motifs is very limited, since their number in tetrapod genomes is relatively small (only 10 proteins in mouse, S1 Table, worksheet 6). Our results contribute to a better understanding of this atypical group of KRAB domain proteins by showing that having two KRAB motifs does not necessarily increase the repressive activity of KRAB-ZNF proteins, but that a cooperative effect of KRAB motifs in binding TRIM28 might be evolutionarily advantageous and therefore may have been an important selective pressure contributing to the evolution of Zinc Finger proteins with more than one KRAB motif.

Our finding that the two KRAB motifs of ZFP568 have very different levels of transcriptional repressive activity motivated us to delve deeper into the factors that contribute to this difference. The first and second ZFP568 KRAB motifs do not differ substantially in amino acid residues previously found to be important for transcriptional repression (see S1 Fig for a summary of published results to date; [11–13,25]). However, we found that amino acid differences at three other residues are important determinants of KRAB domain repressive activity. By identifying these new critical residues, our work provides additional clues about the factors that influence KRAB-mediated transcriptional repression.

An important aspect of our studies is that, in contrast to previous reports, which analyzed the effect of non-conservative mutations in KRAB domain residues, our analysis of ZFP568 highlighted differences in the transcriptional repressive activity of KRAB motifs that exist naturally in vertebrate genomes. This realization motivated us to determine whether the mouse genome contains KRAB-ZNF proteins with non-conservative substitutions at critical residues, as well as whether these proteins would be poor transcriptional repressors. Our statistical analysis indicates that approximately 69% of mouse KRAB domain proteins contain more than one non-conservative substitution at critical amino acid residues, and as many as 16% contain more than three non-conservative changes with respect to the consensus (S1 Table, worksheet 4). When a selection of these proteins was tested for transcriptional repressive activity, we found that some of them, as we had anticipated, showed relatively low transcriptional repression. These results raise the question of whether these KRAB domain proteins are functionally relevant in mammalian genomes. KRAB-ZNF genes have been shown to be amongst the fastest evolving genes in mammals, a conclusion supported by their frequent location in large genomic clusters that are prone to duplication, deletion and non-allelic gene conversion [6]. Consequently, KRAB domain proteins with non-conservative substitutions in critical residues might be just by-products of the fast evolutionary rate of this protein family. In this sense, it is worth noting that some of the KRAB domain proteins used in our study are linked to important biological processes and/or disease conditions, including obesity-associated diabetes (ZFP69; [31–33]) and the regulation of the transcriptional repressor Jumonji/Jarid2 (ZFP496; [27]), while others, although still uncharacterized, are differentially expressed in response to certain experimental conditions or developmental stages [34–36]. Therefore, our observations indicate that a considerable percentage of KRAB-ZNF proteins in our genomes might perform important functions despite having lost their ability to repress transcription during the course of evolution.

During our analysis of mouse KRAB domain proteins, we were surprised to find that ZFP112, ZFP61, ZFP819 and ZFP13 showed a relatively high repressive activity despite containing non-conservative amino acid substitutions at critical residues. This result might be due to a weak effect of the particular amino acid substitutions in these proteins. However, we were particularly intrigued by the high repressive activity of ZFP112 and ZFP13, since these proteins showed an impaired ability to recruit TRIM28. This observation is similar to a previous study of human KRAB-ZNF proteins, which identified TRIM28-independent transcriptional repression in KRAB domain proteins containing SCAN motifs [26]. However, proteins in the Itokawa et al. study were shown to repress transcription in a KRAB-independent fashion, a conclusion consistent with the high number of non-conservative substitutions at critical KRAB domain residues in these proteins (S3 Fig). In contrast, we show that the transcriptional repression activity of ZFP13 is partially mediated by the KRAB motif, providing evidence that KRAB-mediated transcriptional repression can be achieved through TRIM28-independent mechanisms.

Overall, our studies provide insight into the mechanisms of KRAB-mediated transcriptional repression by showing that TRIM28 recruitment is neither sufficient, nor required for the transcriptional repressive activity of certain mouse KRAB domain proteins. Additionally, our results raise awareness about the high percentage of KRAB domain proteins in the mouse genome containing amino acid substitutions that can compromise TRIM28 binding and repressive activity. Our findings challenge the assumption that all KRAB domain proteins function as transcriptional repressors and provide information to assess the transcriptional activity of KRAB domain proteins.

## Materials and Methods

### Animal subjects

*Zfp568*^*chato*^ and *Zfp568*^*P103E09*^ embryos were obtained as previously described [28]. All experiments involving mice were done according to Cornell’s standard operating procedures and ethical guidelines as stipulated in our animal protocol (Protocol number 2005–0102), approved by Cornell’s Institutional Animal Care and use Committee (IACUC). Euthanasia was performed using CO_2_ and cervical dislocation. Animal care and euthanasia were performed with considerations to minimize animal suffering.

### Luciferase Assays

HEK293T cells (purchased from ATCC Catalog # CRL-11268) were transfected using Lipofectamine 2000 (Invitrogen) with pGL3-5XUAS Firefly luciferase reporter, a Gal4DBD effector and pRL Renilla luciferase plasmids. Total amount of DNA transfected was held constant by cotransfecting pCMV-MYC as needed. Cells were assayed with the Dual-Luciferase Reporter System (Promega) 24 hours after transfection. For small interfering RNA (siRNA) knockdown, 8 pmol of *Trim28* siRNAs #1 (19779), #2 (19778) or non-silencing siRNA (Ambion) was transfected using Lipofectamine RNAiMax (Invitrogen). Cells were transfected with luciferase effectors and reporters 24 hours after siRNA transfection and luciferase was assayed after another 48 hours. For each luciferase assay, duplicate transfections and replicate lysates were measured for each condition (n = 4). Luminescence was quantified with a luminometer plate reader (BioTek). Firefly luciferase expression was normalized to Renilla to control for transfection efficiency. Fold repression was calculated compared to Gal4DBD. Lysates were analyzed by western blotting (not shown) to ensure consistent protein expression (protein loading was normalized to Renilla expression). Statistical analysis was performed using paired, two-tailed t-test.

### Western Blots

HEK293T cells were transfected with Lipofectamine 2000 (Invitrogen). Western blots were performed using standard protocols and analyzed using the Odyssey Digital Imager (LiCor). The following validated commercially available antibodies were used: rabbit polyclonal anti-TRIM28 (H-300, Santa Cruz Biotechnology; 1:500), mouse monoclonal anti-Flag (M2, Sigma-Aldrich; 1:500–1:700), normal mouse/rabbit IgG (sc-2025 and sc-2027, Santa Cruz Biotechnology), goat anti-mouse and goat anti-rabbit IR secondary 800CW or 680LT dyes (926–32210, 926–32211, 926–68021, 926–68020, LiCor; 1:15,000).

### Yeast two-hybrid assays

Bait Gal4DBD (pGBKT7 DNA-BD) fusion plasmids with wild type or mutant KRAB-ZNF proteins and prey AD (pGADT7 AD) fusion plasmids with TRIM28 were sequentially transformed into AH109 yeast strain as specified in the Matchmaker GAL4 Two-Hybrid System 3 (Clontech). After transfection, yeast were plated in Leu-Trp- media to select for colonies containing bait and prey constructs, then colonies were re-plated to test for protein interactions onto Ade-His Leu-Trp- plates (high stringency media) or Leu-Trp- X-alpha-gal plates (low stringency media).

### Constructs and primers

Plasmids pCDNA3.1-Gal4DBD, pGL35XUAS firefly luciferase and pRL *Renilla* luciferase are described in (Mascle et al., 2007). Other constructs were generated as described in S2 Table.

### Sequence analysis

Analysis of mouse KRAB-ZNF protein sequences was done using the dataset in [37] and regular expressions in Python to identify individual KRAB motifs and amino acid residues (code available upon request). Sequence logos for the mouse dataset were created using the Weblogo application ([38], available online at http://weblogo.berkeley.edu/logo.cgi).

## Supporting Information

S1 Fig Summary of published data about the effects of KRAB motif amino acid substitutions on transcriptional repression, TRIM28 binding and SRY binding. Data were obtained from [11–13,25], as well as from results shown in Fig 5.
(TIF)

S2 Fig Domain structure of the KRAB domain proteins analyzed in this study. (TIF)

S3 Fig KRAB domain protein alignment. Sequence alignments of the consensus KRAB domain sequence [11] with all mouse KRAB domain proteins used in this study **(A)**, as well as the human KRAB-ZNFs analyzed in [26] **(B)**. Residues highlighted in grey indicate amino acids important for repressive activity (as described in [11–13,25]). Additional amino acid residues critical for transcriptional repression as described in this study are highlighted in yellow (see Fig 5). Red font was used to highlight critical KRAB residues that constitute non-conservative amino acid substitutions with respect to the KRAB consensus sequence and/or are not highly represented in KRAB domain proteins.
(TIF)

S1 Table Analysis of mouse KRAB domain proteins. The 357 mouse KRAB domain proteins identified in the Corsinotti et al. 2013 study [1] were sorted according to their chromosomal location (see worksheet #2) or the conservation of their KRAB motif at critical amino acid residues important for KRAB-mediated transcriptional repression and/or TRIM28 binding (see worksheet #3). Statistics about the % of KRAB domain proteins with non-conservative substitutions at these critical residues is provided in worksheet #4. Worksheet #5 contains a summary of the non-conservative amino acid substitutions found at each critical residue. Lists of proteins with 2 KRAB motifs, SCAN domains and without ZNF motifs (KRAB-O) are provided in Worksheets #6 to 8. Proteins used in this study are highlighted in red. For proteins containing two KRAB motifs, “_K1” and “_K2” suffixes were used to identify the first and second KRAB domains, respectively.
(XLSX)

S2 Table Constructs and primers used. (XLSX)
