# Supplementary figures and images for: The Transcriptional Repressive Activity of KRAB Zinc Finger Proteins Does Not Correlate with Their Ability to Recruit TRIM28
Source: PLoS One. 2016 Sep 22;11(9):e0163555. doi: 10.1371/journal.pone.0163555 (PMC5033580; doi:10.1371/journal.pone.0163555)

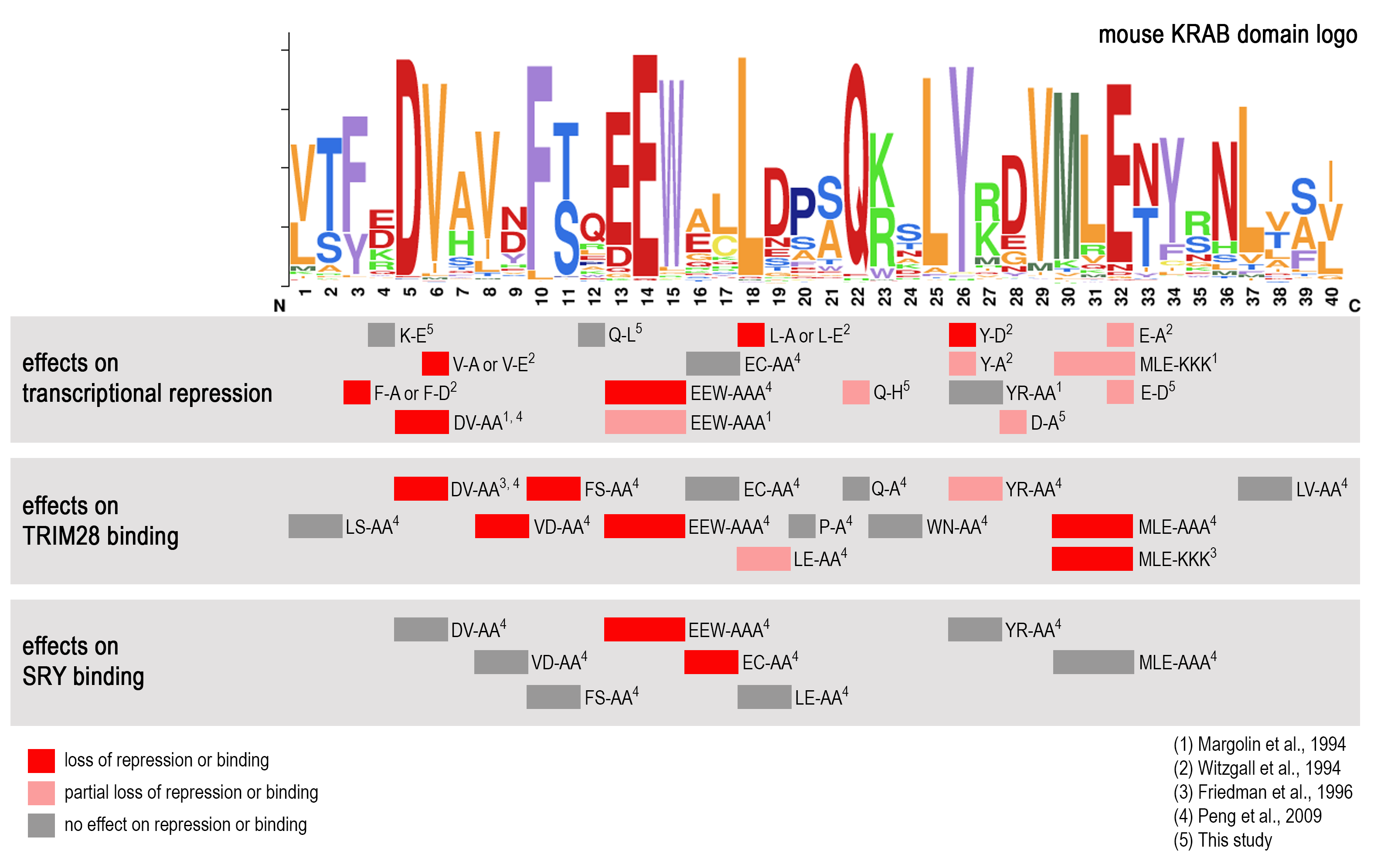

Supplement: S1 Fig — Data were obtained from [11–13,25], as well as from results shown in Fig 5. (TIF) [file pone.0163555.s001.tif]

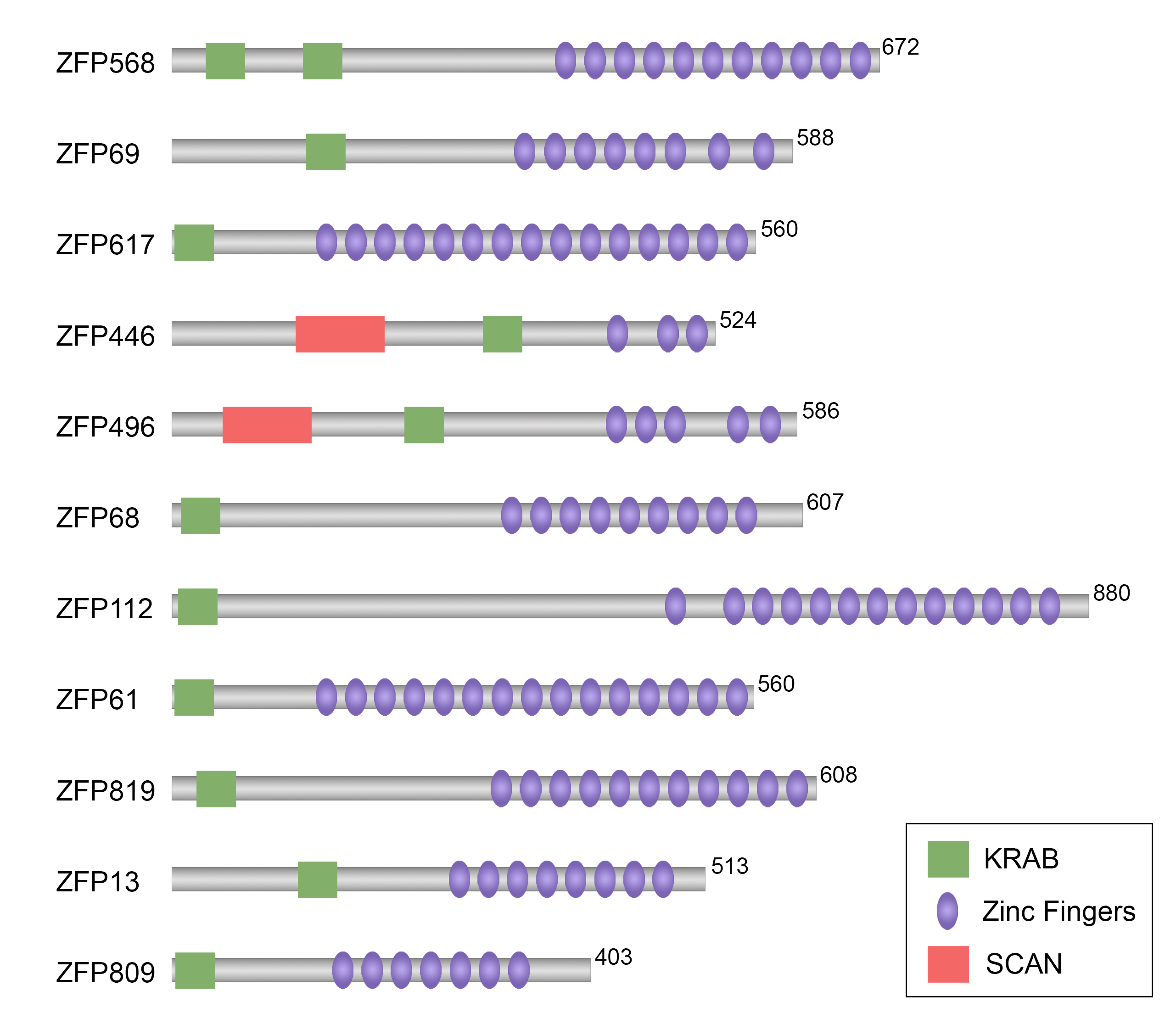

Supplement: S2 Fig — (TIF) [file pone.0163555.s002.tif]

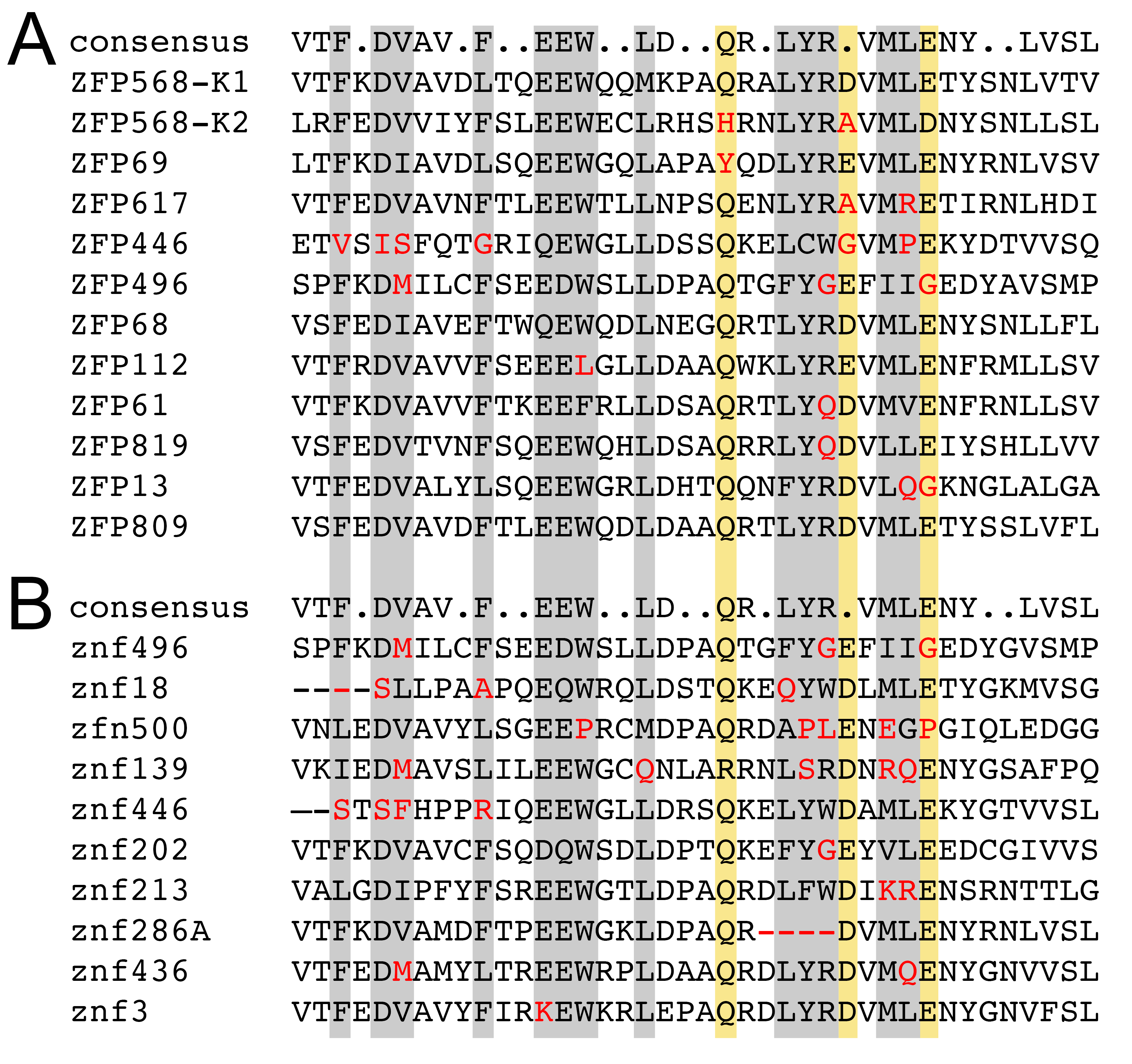

Supplement: S3 Fig — Sequence alignments of the consensus KRAB domain sequence [11] with all mouse KRAB domain proteins used in this study (A), as well as the human KRAB-ZNFs analyzed in [26] (B). Residues highlighted in grey indicate amino acids important for repressive activity (as described in [11–13,25]). Additional amino acid residues critical for transcriptional repression as described in this study are highlighted in yellow (see Fig 5). Red font was used to highlight critical KRAB residues that constitute non-conservative amino acid substitutions with respect to the KRAB consensus sequence and/or are not highly represented in KRAB domain proteins. (TIF) [file pone.0163555.s003.tif]
